# The Effect of Group Reminiscence on the Cognitive Status of Elderly People Supported by Ilam Welfare Organization in 2013; A Randomized Controlled Clinical Trial

**Published:** 2014-10

**Authors:** Iran Jahanbin, Sara Mohammadnejad, Farkhondeh Sharif

**Affiliations:** 1 Community Based Psychiatric Care Research Center, Department of Community Health Nursing, School of Nursing and Midwifery, Shiraz University of Medical Sciences, Shiraz, Iran;**; 2 Department of Community Health Nursing, School of Nursing and Midwifery, Shiraz University of Medical Sciences, Shiraz, Iran;**; 3 Community Based Psychiatric Care Research Center, Department of Mental Health and Psychiatric Nursing, School of Nursing and Midwifery, Shiraz University of Medical Sciences, Shiraz, Iran

**Keywords:** Cognitive Status, Elderly, Reminiscence.

## Abstract

**Background: **Cognitive impairments, which are common problems among the elderly people, account for a wide range of aging disorders. Group reminiscence can be used as a profitable therapeutic method for preventing cognitive-behavioral disorders in older adults. Therefore, we aimed to investigate the effect of group reminiscence on the cognitive status of elderly people.

**Methods: **This study was a non-blinded randomized controlled trial. We enrolled 100 elderly people who were under the support of Ilam Welfare Organization, western Iran in 2013. Balanced block randomization method was used to randomize the participants into intervention and control groups.Elderly people in the intervention group participated in a group reminiscence program consisted of two one-hour sessions per week for 8 consecutive weeks. Data were collected using Mini Mental State Examination (MMSE). The questionnaire was completed four times by the participants; before, immediately after, two and three months after the intervention.

**Results: **The mean±SD of cognitive status scores in the intervention group was 24.66±3.8 which increased to 25.02±3.67, 25.04±3.72 and 24.72±3.66 immediately after, two and three months after the intervention respectively. The results showed that the changes in the mean scores were statistically significant in the intervention group immediately after the intervention (P=0.001) and at second month (P=0.003) follow-ups. However, we found no statistically significant difference in the intervention group at the mentioned time intervals in this regards (P=1.000).

**Conclusion: **We concluded that continuous programs of group reminiscence could improve cognitive status of elderly population.

**Trial Registration Number: **IRCT201405147531N7

## Introduction

Ageing is one of the greatest population changes in the 20^th^ century. This phenomenon is predicted to cause the number of elderly to increase from 600 million in 2000 to 2 billion by 2050.^1^ Population ageing is known as a consequence of the demographic transition in which fertility and mortality reduce from high levels to low ones.^2^

Median age in Iran which was 21.1 years in 1950 and 23.4 years in 2005, will reach to 40.6 years in 2050.^3^Considering increasing elderly population in Iran, special attention should be paid to the needs of this segment of community of which the need for health is one of the most important ones. Health is a fundamental right of every human being and also a social goal so that all governments must provide the health of the community.

Biological functions progressively decline with aging and elderly people gradually lose their physiological, psychological and social functioning. Changes in the disease pattern, which were followed by reduction of infectious diseases and increase of longevity and chronic diseases, had drawn more attention towards the concepts of health and life improvement over the past decades.^4^

Furthermore, cognitive impairments are common problems in the elderly people. They account for a wide range of aging disorders so that 35% of elderly show varying degrees of such impairments of which Alzheimer’s disease is a progressive one. Accordingly, considering the health status of such age group, for improving their health, preventing diseases, and planning for care, seems necessary.^5^

However, changes in cognitive functioning may be seen as an early sign before the occurrence of behavioral manifestations. Therefore, those older people who are at risk should be identified and be under consideration to be protected from cognitive defects and disabilities resulted from life expectancy reduction. Consequently, their needs for health care supplies will decrease as well.^6^

Spalding and Sebesta (2008) described Alzheimer as the sixth prominent cause of mortality in people aged 65 and over in United States in 2002, and there is no program for screening.^7^ Simon et al. (2012) confirmed that cognitive therapy could improve and reinforce cognitive functioning and memory in people with dementia.^8^

Cognitive impairments can increase the risk of developing Alzheimer’s disease in older adults if not diagnosed and managed early. Several treatment methods are used to overcome cognitive impairments in older adults. Pharmaceutical therapies are less welcomed due to their side effects. 

Reminiscence is a non-pharmaceutical therapy and a form of therapeutic intervention which is often used for older people. In the classifications of interventions and nursing care, it is described as recalling past events, feelings and thoughts in order to create and facilitate the feeling of pleasure, enhance the quality of life, and adopt with the current situation.^9^ Reminiscence therapy, which is derived from Ericson’s theory, helps elderly people review their past. Thus, it helps individuals to maintain the integrity of their characters.^10^^,^^11^

Accordingly it seems that considering psychological and cognitive problems associated with old ages is essential due to the sensitivity of such critical period of human life. On other hand, using simple and available solutions can be helpful in the improvement of mental health and cognitive impairments. 

The importance of reminiscence therapy for older adults and the role of such intervention in improving their depression have been examined and confirmed by several studies; however, only a few studies in Iran have addressed such issue. Since the studies which are conducted in Iran often conducted without control group and follow-up, we aimed to investigate the effect of group reminiscence on cognitive status of elderly people. 

## Patients and Methods

**Figure 1:**
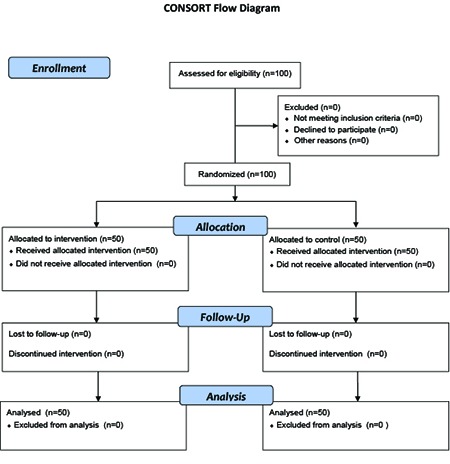
CONSORT flow diagram of the participants

This study was a non-blinded randomized controlled trial and was approved by the Ethics Committee of Shiraz University of Medical Sciences. We enrolled 100 elderly people who were under the support of Ilam Welfare Organization, western Iran during July to August 2013. The patients were selected using a simple sampling method. A balanced block randomization method was used to randomize the participants, who met inclusion criteria, into intervention (n=38) and control groups (two groups of 50 each) (figure 1).

The sample size was calculated as 50 in each group based on the data of similar study conducted by Akhondzadeh et al. (2012) and using (Med Calc) software (power: 80%, α: 0.05, and SE: 40%).^12^

$$n^{´}=\frac{{\left(z_{1-\frac{\alpha }{2}}+z_{1-\beta }\right)}^{2}}{{\left(\frac{\text{d}}{2\delta }\right)}^{2}}$$

$$\left(n_{1}=n_{2}\right)$$

Inclusion criteria included willingness to participate in the study, no history of severe psychological disorders and hospitalization in the previous three months as well as ability to communicate visually and verbally and participate in the reminiscence classes. But the participants scored lower than 10 points on Mini Mental State Examination (MMSE) scale were excluded from the study.  

After obtaining informed consent, data were collected using demographic questionnaire and the brief 30-item MMSE questionnaire. Demographic data included age, sex, educational and occupational status and place of residence.

MMSE scale is one of the most commonly used instruments for screening cognitive functioning and assessing the domains of cognitive functions including orientation, registration, attention, calculation and memory recall. Obtainable scores on the MMSE range from 0 to 30, whereby a score of lower than 25 indicates the likelihood of cognitive impairment. Scores of 21-24 are considered as mild, 10-20 as moderate and <10 as severe impairment.^13^

Validity and reliability of the questionnaire has been confirmed by Frooghian and colleagues using Cronbach’s alpha. Sensitivity, specificity and Cronbach’s alpha were calculated as 78%, 93% and 89% respectively for the Farsi version ^14^ The questionnaire was completed four times by the participants; before, immediately after, two and three months after the intervention.

Elderly people in the intervention group were divided into 5 groups of 10 people to ensure that everyone could have an equal chance of being trained successfully. Each group participated in a group reminiscence program consisted of two one-hour sessions per week for 8 consecutive weeks. All the sessions were conducted and managed by researcher. The reminiscence sessions included recalling and sharing participants’ memories of childhood, adulthood, education, marriage, birth of children, work experience, achievements, trips, celebrations and special past events in their life. However, the elderly participants of the control group received no intervention.  

After the end of all sessions, the questionnaires were completed again by the people in both groups immediately after, two and three months after the intervention.

The collected data were analyzed using SPSS software, version 16. Statistical descriptive tests such as mean and standard deviation (SD) and percentage were used to describe the features of the data. Analytical statistical tests such as independent t-test, repeated measures analysis of variances (ANOVA) and Bonferroni - hoc tests were used as appropriated. The significance level was set values lower than <0.05. 

## Results

The mean±SD age of the participants was (70.96±8.07). According to table 1 two groups were similar regarding demographic variables. The mean±SD of cognitive status scores were 24.66±3.8 and 24.86 ±3.52 in the intervention and control group respectively before the intervention.

**Table 1:** Frequency distribution of demographic variables in the intervention and control group

**Variables**		**Intervention Group** **Frequency (Percentage)**	**Control Group** **Frequency (Percentage)**	**P value**
Sex	Men	29 (58)	29 (58)	1
	Women	21(42)	21 (42)	
Marital Status	Single	4 (8)	5 (10)	1
	Married	34 (68)	33 (66)	
	Widow	12 (24)	12 (24)	
Occupational status	Farmer	12 (24)	13 (26)	0.522
	Clerk	6 (12)	9 (8)	
	Retired	15 (30)	9 (18)	
	Housewife	17 (34)	19 (38)	
Place of Residence	Urban	31 (62)	29 (58)	0.683
	Suburban	31 (62)	29 (58)	
Educational status	Illiterate	35 (70)	34 (68)	0.114
	Primary Education	4 (8)	10 (20)	
	Secondary Education	4 (8)	0 (0)	
	High School Diploma	6 (12)	4 (8)	
	Higher Education	1 (2)	2 (4)	

The mean±SD scores in the intervention group increased to 25.02±3.67 and 25.04±3.72 after two and three months respectively. Similarly, the mean±SD scores in the control increased to 24.98±3.52, 25.00±3.50, and 24.94±3.51 immediately after, two and three months respectively.  

The results confirmed that the changes in the mean scores were statistically significant in the intervention group immediately after the intervention (P=0.001) and at the second month (P=0.003) follow-ups. However, we observed no significant difference at the third month follow-up after the intervention (P>1.00).In the control group, we also found no statistically significant difference at the mentioned time intervals in this regards (P>1.00), (table 2).

**Table 2:** Comparing the mean (±SD) of MMIE in intervention and control groups before and after the intervention

**Variable**	**Time**	**Before Intervention**	**Immediately after the Intervention**	**Two month after the Intervention**	**Three months after the Intervention**
	**Group**	**Mean±SD**			**Adjusted mean±SD**
MMSE score	Intervention Group	24.66±3.8	25.02±3.68	25.04±3.72	24.72±3.66
	Control Group	24.86±3.52	24.98±3.52	25.00±3.50	24.94±3.51
P value			0.003	0.001	1

*Significant differences at α=0.05

## Discussion

The mean±SD changes of cognitive status showed no significant differences between the intervention and control groups in terms of demographic characteristics. The results also indicated that the difference in cognitive status scores of the two groups is not correlated with any of the demographic variables. Our findings are similar to those of some studies,^15^^,^^16^ and in contrast with some others.^17^^,^^18^ Hence, we can conclude that the both groups were homogeneous in terms of population and demographic structures.

There was an increase in cognitive status scores of elderly people in the intervention group immediately after and two months after the intervention as compared with before the intervention. But, the scores of cognitive status did not increase at the third month after the intervention in comparison with other assessment time points (24.72±3.66). However, we observed no significant difference in the control group in this regards. The estimated recovery percentage and effect size, in the studies conducted on cognitive interventions for elderly people, confirmed the effectiveness of the applied interventions in the cognitive functioning of the intervention group. 

Our finding, proving the effects of cognitive interventions such as reminiscence on cognitive impairments of older adults, was consistent with that of Beck et.al (1998) who examined the effect of such interventions on cognitive status of elderly people age 60-98 during 6 weeks. Their findings showed that the cognitive status scores after the intervention were higher than those of before the intervention.^19^

The results of the several studies revealed that cognitive intervention could improve cognitive scores in old age.^20^^-^^27^ Similarly, McKrracher et al. concluded that cognitive rehabilitation could significantly improve cognitive status of senior citizens.^28^

Besides, Olinde (2006) observed that using rehabilitation interventions such as training elderly to learn the names and the pictures of countries can cause memory retrieval and prevent distraction in those with probable cognitive impairments.^29^ Likewise, Stuss et al suggests that cognitive rehabilitation in older people can serve as an appropriate solution for reinforcing the memory and improving mental status. Similar to our findings, the results of aforementioned studies specified that cognitive interventions can improve and enhance cognitive scores in elderly population.^30^ Therefore we can also conclude that cognitive interventions such as reminiscence can effectively improve the cognitive score of older people.

Nemati, Dehkordi et al. reported that group reminiscence helps to stimulate the elderly’s memory and reinforce it through reducing anxiety and improving cognition.^31^Amini et al. also confirmed that cognitive rehabilitation techniques could improve cognitive functioning and memory.^32^ Likewise, the findings of Aquirra et al. shows that reminiscence therapy is an effective therapeutic approach which can improve cognitive functioning and reinforce the effects of other therapies in elderly adults.^33^

Woods showed that reminiscence prevents the degradation of cognitive functioning by stimulating memory. He considered this intervention as an effective treatment for improving and rehabilitating patients suffering from dementia.^34^ Moreover, Joosten et al. believes that cognitive therapy leads to increase of awareness, memory, attention, and motivation and decrease of behavioral problems. Consequently, using such method the patients with cognitive impairments will require less care.^35^ One study reports that reminiscence is an effective method of therapy which can reduce cognitive-behavioral impairments and alleviate physiological symptoms as well and another study emphasized on the effect of such therapy on cognitive status.^36^^,^^37^

We also observed that cognitive scores reduced in the intervention group three months after the intervention. In the most studies conducted on cognitive rehabilitation of elderly people, the effect of treatment interventions on the elderly’s cognitive status mostly started after 8 to 10 sessions.^38^^-^^40^The studies also proved that sustained effects could be only achieved through continuation of therapeutic intervention. We also observed the effects of intervention after 8 sessions.

Moreover, the changes in the mean±SD were not statistically significant at the third month follow-up indicating the discontinuity of the intervention. Therefore, the continuous use of cognitive therapy could be helpful for elderly people over time. 

A limitation of the study was our relatively small sample size. Therefore, our findings cannot be generalized to a broader community. The researchers suggest further studies with a larger sample size to be carried out on a larger statistical community. Moreover, most of our participants were in the same age range and had similar income and educational level. Hence, there was no possibility to examine the effect of such variables on our elderly population. 

Furthermore, we could not continue the group reminiscence program for more than 8 weeks due to the lack of long-term cooperation of the participants. Consequently, conducting studies with an intensive and a long-term reminiscence intervention are highly recommended for better evaluation of the therapy’s effect on the improvement of cognitive status among senior citizens. 

## Conclusion

We conclude that group reminiscence could be used as a simple, cost effective and applicable therapy by health care professionals, care providers and families to improve cognitive status in older adults. It can also help to prevent common problems associated with aging, such dementia and Alzheimer’s disease, which could result in psychological problems for elderly people and their families. Moreover, considering the side effects of medications used for the treatment of cognitive impairments and attempts to minimize the use of medications in elderly people, reminiscence therapy could be used as an appropriate alternative for pharmaceutical therapies of such age group. 
